# Improving the Cellulose Enzymatic Digestibility of Sugarcane Bagasse by Atmospheric Acetic Acid Pretreatment and Peracetic Acid Post-Treatment

**DOI:** 10.3390/molecules28124689

**Published:** 2023-06-10

**Authors:** Yuchen Bai, Mingke Tian, Zhiwei Dai, Xuebing Zhao

**Affiliations:** 1 China Food Flavor and Nutrition Health Innovation Center, Beijing Technology and Business University, Beijing 100048, China; baiyuchen@btbu.edu.cn (Y.B.);; 2 Beijing Key Laboratory of Flavor Chemistry, Beijing Technology and Business University, Beijing 100048, China; 3 Key Laboratory of Industrial Biocatalysis, Ministry of Education, Tsinghua University, Beijing 100084, China; 4 Institute of Applied Chemistry, Department of Chemical Engineering, Tsinghua University, Beijing 100084, China

**Keywords:** sugarcane bagasse, lignocellulosic biomass, pretreatment, acetic acid delignification, kinetic modeling, peracetic acid post-treatment

## Abstract

Pretreatment of sugarcane bagasse (SCB) by aqueous acetic acid (AA), with the addition of sulfuric acid (SA) as a catalyst under mild condition (<110 °C), was investigated. A response surface methodology (central composite design) was employed to study the effects of temperature, AA concentration, time, and SA concentration, as well as their interactive effects, on several response variables. Kinetic modeling was further investigated for AA pretreatment using both Saeman’s model and the Potential Degree of Reaction (PDR) model. It was found that Saeman’s model showed a great deviation from the experimental results, while the PDR model fitted the experimental data very well, with determination coefficients of 0.95–0.99. However, poor enzymatic digestibility of the AA-pretreated substrates was observed, mainly due to the relatively low degree of delignification and acetylation of cellulose. Post-treatment of the pretreated cellulosic solid well improved the cellulose digestibly by further selectively removing 50–60% of the residual linin and acetyl group. The enzymatic polysaccharide conversion increased from <30% for AA-pretreatment to about 70% for PAA post-treatment.

## 1. Introduction

Sugarcane bagasse (SCB) is a fibrous matter that remains after sugarcane is crushed to extract juice. It is an abundant lignocellulosic biomass with a global production of more than 100 metric tons [1]. Various applications of SCB have been developed for producing chemicals, fuels, and materials [2,3]. One of the most promising utilizations of SCB is to produce second-generation bioethanol, because SCB has a relatively high cellulose content, having low ash content [4]. However, being similar to other lignocellulosic feedstock, SCB has to be pretreated prior to converting the cellulose to ethanol in order to increase the accessibility of cellulose towards cellulase enzymes for improving saccharafication efficiency. Various pretreatments have been employed to increase the cellulose digestibility of SCB, such as dilute acid [5], steam explosion [6], alkaline pretreatment [7], and ionic liquid pretreatment [8,9]. After pretreatment, the saccharafication efficacy of SCB can be increased to 70–90%. Organosolv pretreatment is a promising pretreatment technique to improve the digestibility of SCB [10,11]. This process employs various organic solvents, with or without the addition of exogenous catalysts at elevated temperatures, to remove a considerable part of lignin, as well as hemicelluloses, thus exposing cellulose [12]. Among the frequently used organic solvents, organic acids, such as acetic acid (AA), show some merits in biomass pretreatment [13]. First, AA has a strong hydrogen bonding ability with a Hildebrand solubility parameter (δ_1_) of 25.8 (J/cm^3^)^−1/2^ close to that of lignin (δ_2_ = 22.5 (J/cm^3^)^−1/2^), and, therefore, it is a good solvent to lignin [14]. Second, the delignification process can be performed under atmospheric pressure when additional mineral acids (H_2_SO_4_ or HCl) are used as catalysts [15]. Third, the AA formed by hydrolysis of the acetyl group in hemicelluloses can be a supplement of the loss AA during pretreatment. Fourth, the H^+^ dissociated from AA can facilitate hemicellulose hydrolysis and lignin fragmentation, thus achieving a fractionation of lignocellulosic biomass. Actually, AA has been used for delignification of lignocellulosic biomass with or without addition of mineral acids for producing pulps from various lignocellulosic biomass feedstocks since the middle of last century [16,17]. However, much less attention has been paid to AA pretreatment of biomass, mainly because acetylation of cellulose takes place during AA delignification, which limits the recognition of cellulose substrates by cellulases [18]. Post-treatment of AA delignified substrates with a small amount of alkali has been found to well remove acetyl group and recover cellulose digestibility [19]. Nevertheless, alkaline deacetylation requires a washing step after AA pretreatment, which would lead to high water consumption. Moreover, the AA pretreatment parameters have to be re-optimized with consideration to acetylation and cellulose enzymatic digestibility. On the other hand, oxidative pretreatment with various oxidants pertains to be an effective method to remove lignin to achieve significantly increased cellulose digestibility. Various oxidative pretreatments have been developed, including wet oxidation, alkaline hydrogen peroxide, organic peracids, Fenton oxidation, and ozone oxidation [20]. After oxidative pretreatment, a considerable part of lignin could be removed with change in cell wall structure, thus greatly exposing cellulose to cellulase enzyme. The oxidative modification of lignin also can change its surface properties, including hydrophobility and surface charges, which might reduce the non-productive adsorption of enzymes on the lignin matrix. However, the direct use of oxidants for pretreatment might be too costly because a relatively large amount of oxidants is needed. Thus, a combination of a delignification process prior to oxidative treatment would reduce the amount of oxidants. Therefore, the objective of the present work is to optimize the AA pretreatment process, considering several response variables, including solid recovery degree of delignification, hemicelluloses (xylan removal), glucan recovery, acetyl group and cellulose digestibility, and investigation of the pretreatment kinetics in terms of total solid solubilization, delignification, and carbohydrate (holocellulose) solubilization. Peracetic acid (PAA) post-treatment was further employed to remove the residual lignin and the acetyl group, which avoids using a washing step after AA delignification, which can reduce the amount of PAA for delignification.

## 2. Results and Discussion

### 2.1. Effects of AA Pretreatment on Chemical Compositions

The CCD experimental and model predicted results are shown in Table 1. Corresponding three-dimensionsal response surface plots are shown in Supplementary Figures S1–S4, and the regressed quadratic polynomial models are shown as Equations (1)–(4). Statistical analysis (ANOVA) (Supplementary Tables S1–S4) indicated that Equation (1) was very significant (*p* = 0.0004 < 0.01) to predict the experimental data. The *p*-values for *T*, *C_AA_*, *C_SA_*, and *t* were very small (<0.01), indicating that these variables showed very significant effects on *SY*. The *p*-value for X_1_^2^ was less than 0.05, indicating that temperature also had a significant non-linear influence on *SY*. As illustrated in Figure S1, *SY* decreased with an increase in the levels of these variables. The decrease in *SY* was mainly due to the removal of lignin and carbohydrate (holocellulose). For *DD*, statistical analysis results (Supplementary Table S2) showed that *T*, *C_AA_*, and *C_SA_* had very significant effects, while *t* had no significant effect. This was mainly because most of the lignin was removed within the first two hours, while the residual lignin demonstrated much lower reactivity [15]. Therefore, prolonging pretreatment time showed no significant increase in *DD* (Supplementary Figure S2). Similarly, temperature also showed a significant non-linear influence on *DD*, while strong interactive effects were found between *T* and *C_AA_*, as well as *T* and *C_SA_*. As shown in Figure S3 and Table S3, all of these four variables showed very significant effects on *HS*, and *HS* increased with the levels of the variables. The solubilization of holocellulose was mainly attributed to solubilization of hemicelluloses (xylan) because hemicelluloses are much more susceptible than cellulose. It was found that cellulose solubilization during AA pretreatment was generally less than 20%, while xylan solubilization could be higher than 80%, depending on pretreatment conditions. Temperature had a significant impact on *HS* because the hydrolysis of the glucosidic bond was greatly temperature-dependent. The hydrolysis of polysaccharide was catalyzed by H^+^, and, therefore, sulfuric acid demonstrated a very significant effect. The effects of AA concentration on *HS* can be attributed to two aspects. First, AA can dissociate H^+^ as a supplemental catalyst for carbohydrate hydrolysis. Second, AA works as a solvent to dissolve the lignin fragments and facilitate the delignification reaction, and removing lignin can expose more carbohydrate. Therefore, apparently, *C_AA_* demonstrated very significant effects on *HS*.

Y_SY_ (%) = 79.28 − 6.77X_1_ − 3.77X_2_ − 2.48X_3_ − 6.05X_4_ − 1.25X_1_X_2_ − 0.77X_1_X_3_ − 1.28X_1_X_4_ + 0.013X_2_X_3_ − 1.36X_2_X_4_ − 0.16X_3_X_4_ − 1.71X_1_^2^ − 0.48X_2_^2^ + 0.34X_3_^2^ − 0.29X_4_^2^ (1)

Y_DD_ (%) = 36.74 + 8.67X_1_ + 5.54X_2_ + 0.84X_3_ + 7.84X_4_ + 2.60X_1_X_2_ − 0.14X_1_X_3_ + 4.36X_1_X_4_ + 0.87X_2_X_3_ + 1.37X_2_X_4_ + 1.80X_3_X_4_ + 2.80X_1_^2^ + 0.52X_2_^2^ + 0.40X_3_^2^ − 0.32X_4_^2^ (2)

Y_HS_ (%) = 22.61 + 7.30X_1_ + 3.81X_2_ + 3.03X_3_ + 6.54X_4_ + 0.85X_1_X_2_ + 0.84X_1_X_3_ + 0.76X_1_X_4_ − 0.57X_2_X_3_ + 0.73X_2_X_4_ + 0.39X_3_X_4_ + 0.10X_1_^2^ − 0.14X_2_^2^ − 1.35X_3_^2^ − 1.53X_4_^2^ (3)

Y_AGC_ (%) = 4.91 − 0.18X_1_ + 0.25X_2_ − 0.074X_3_ − 0.043X_4_ + 0.024X_1_X_2_ − 0.045X_1_X_3_ − 0.15X_1_X_4_ + 0.040X_2_X_3_ + 0.061X_2_X_4_ − 0.062X_3_X_4_ − 0.093X_1_^2^ + 0.030X_2_^2^ − 0.038X_3_^2^ − 0.19X_4_ (4)

Acetylation of cellulosic solid took place during *AA* pretreatment. This was mainly caused by the esterification reaction between cellulose hydroxyl groups and *AA*. However, the tendency of effects of *T*, *C_AA_*, *C_SA_*, and *t* on *AGC* were somewhat different from those on *SY*, *DD* and *HS*. There was a maximal value for *AGC* depending on the pretreatment condition, and only temperature and *AA* concentration showed very significant impacts (Supplementary Table S4). It can be known, from Figure S4, that *AGC* increased with *AA* monotonically, while parabolic tendency was observed for *T*, *C_SA_*, and *t*. This is because hemicellulose contains an acetyl group, and deacetylation also takes place with solubilization of hemicelluloses during *AA* pretreatment. As the removal of hemicelluloses and lignin increased, more cellulose was exposed, and acetylation of cellulose became significant. Therefore, the maximal *AGC* was dependent on the kinetic rates of hemicellulose deacetylation and cellulose acetylation.

**Figure 1:**
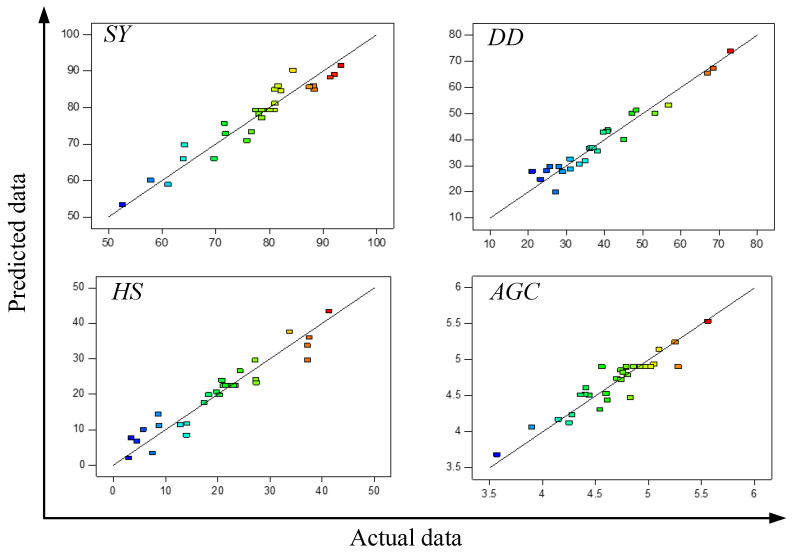
Comparison of actual experimental data and quadratic polynomial model-predicted data for *SY*, *DD*, *HS*, and *AGC*. The red color indicates a high level of the response variable, while the blue color indicates a low level.

**Figure 2:**
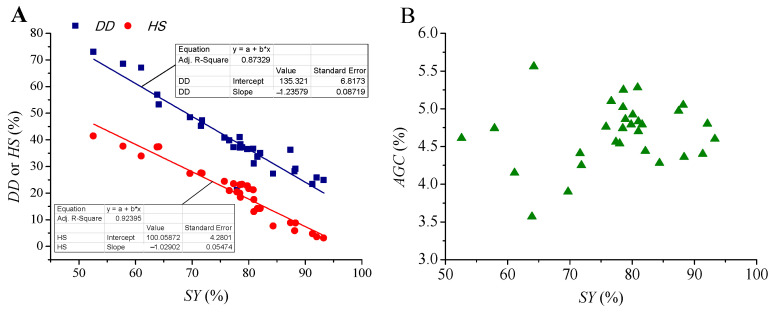
Plots of *SY* with *DD*, *HS*, or *AGC*. (**A**): *SY* with *DD* and *HS*; (**B**): *SY* with *AGC*.

The determination coefficients (*R*^2^) of the quadratic polynomial models for *SY*, *DD*, *HS*, and *AGC* (Equations (1)–(4) were in the range of 0.9173, 0.9484, 0.9142, and 0.8614, respectively. Plots of actual data with the model predicted data shown in Figure 1 indicated that these models were accurate enough to predict most of the experimental data. Further analysis showed that linear relationships were found between *SY* and *DD*, as well as *SY* and *HS*, with *R*^2^ of 0.8733 and 0.9240, respectively (Figure 2). Thus, *DD* and *HS* can be roughly estimated by *SY*, since *SY* was much easier to determine. However, no apparent mathematical relationship was found between *SY* and *AGC*.

### 2.2. Kinetics of Delignification and Solubilization of Holocellulose

In order to further understand the kinetic behaviours of AA pretreatment, the kinetics of total solid solubilization (*S_S_*), degree of delignification (*D_d_*), and holocellulose solubilization (*H_S_*) were investigated using 75% AA with an addition of 0.1–0.4% SA at different temperatures. Both Saeman’s model and the “potential degree of reaction (PDR)” model were considered to kinetically simulate the AA pretreatment process.

#### 2.2.1. Saeman’s Model

Saeman’s model is the simplest kinetic model to describe biomass hydrolysis [21]. Taking delignification as an example, this model considers the rate of lignin solublization is a pseudo-homogeneous first-order reaction, with the residual (unreacted) lignin fraction defined as follows:

(5) $$-\frac{dC_{L}(t)}{dt}=k_{L}C_{L}(t)$$

where; *C_L_*(*t*) is the lignin concentration (g/L) in the pseudo-homogenous reaction system at time *t*. One can define the degree of delignification (*D_d_*) at time *t* as the following equation:

(6) $$D_{d}(t)=\frac{C_{L0}-C_{L}(t)}{C_{L0}}$$

where; *C_L_*_0_ is initial lignin concentration (g/L) in the pseudo-homogenous reaction system, and we, thus, also can describe the rate of xylan solubilization as:

(7) $$\frac{dD_{d}}{dt}=k_{L}(1-D_{d})$$

with; *D_d_*(0) = 0. The integral form of Equation (7) is:

(8) $$D_{d}=1-\exp(-k_{D}t)$$

where; the value of *D_d_* is in the range of 0–1, and *k_L_* is the rate constant. Therefore, the rate constant can be determined by ploting ln(1 − *D_d_*) with *t*. Similarly, the integral forms of Saeman’s models for total solid and holocellulose solubilizations are:

(9) $$S_{S}=1-\exp(-k_{S}t)$$

(10) $$H_{S}=1-\exp(-k_{H}t)$$

where; *S_S_* and *H_S_* are ratio of total solid solubilization and degree of delignification, respectively; *k_S_* and *k_H_* are corresponding rate constants, which can be determined by plotting ln(1 − *S_S_*) and ln(1 − *H_S_*) with *t*, respectively. According to experimental data, the plots of ln(1 − *S_S_*), ln(1 − *D_d_*) and ln(1 − *H_S_*) with *t* are shown in Supplementary Figures S5–S7. However, as shown in these figures, ln(1 − *S_S_*), ln(1 − *D_d_*), and ln(1 − *H_S_*) actually had apparent deviation from linear relation. The determination coefficients (*R*^2^) were in the range of 0.46–0.98, with most of them being less than 0.6. It indicated that AA-pretreatment did not follow the kinetics described by Saeman’s model.

#### 2.2.2. The “Potential Degree of Reaction (PDR)” Model

The “Potential degree of reaction (PDR)” model was developed by Zhao et al. to describe the kinetic behaviors of dilute acid [22] and organosolv pretreatments [15,23]. This model has been found to fit well with different chemical pretreatments of various biomass feedstocks [24]. The PDR model was developed based on the multilayered structure of the plant cell wall and heterogeneity of the reaction system. The biomass component distributed in different layers of the cell wall showed different reactivity, depending on the reaction severity. Thus, a parameter representing the potential degree of reaction, such as potential degree of delignification, was introduced into the kinetic model. In the present work, potential degree of total solid solubilization (*δ_SS_*), delignification (*δ_DD_*), and holocellulose solubilization (*δ_HS_*) were proposed. The PRD models for total solid solubilization, delignification, and holocellulose solubilization, thus, can be expressed as Equations (11)–(13), with integral forms shown as Equations (14)–(16), respectively.

(11) $$\frac{dS_{S}}{dt}=k_{S}(\delta_{SS}-S_{S})$$

(12) $$\frac{dD_{d}}{dt}=k_{L}(\delta_{DD}-D_{d})$$

(13) $$\frac{dH_{S}}{dt}=k_{H}(\delta_{SS}-H_{S})$$

(14) $$S_{S}=\delta_{SS}\left[1-\exp(-k_{S}t)\right]$$

(15) $$D_{d}=\delta_{DD}\left[1-\exp(-k_{D}t)\right]$$

(16) $$H_{S}=\delta_{HS}\left[1-\exp(-k_{H}t)\right]$$

**Figure 3:**
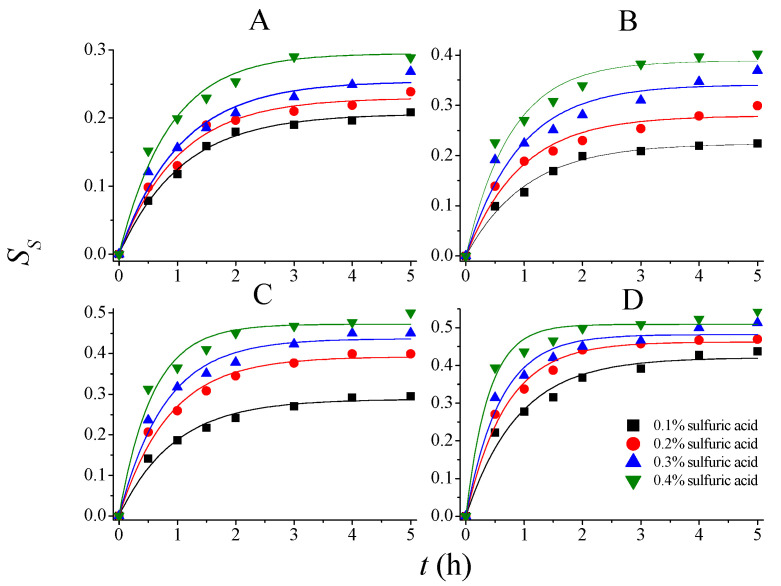
Experimental and model-predicted kinetic data for total solid solubilization at different temperatures. (**A**): 80 °C; (**B**): 90 °C; (**C**): 100 °C; and (**D**): 110 °C.

**Figure 4:**
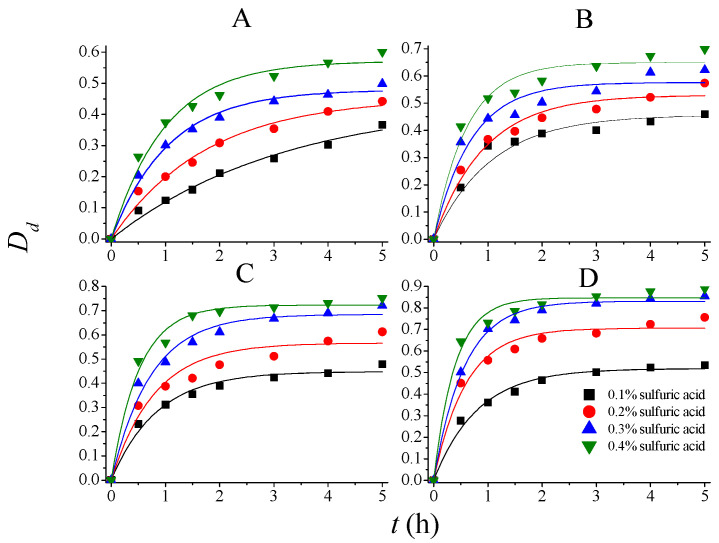
Experimental and model-predicted kinetic data for degree of delignification at different temperatures. (**A**): 80 °C; (**B**): 90 °C; (**C**): 100 °C; and (**D**): 110 °C.

**Figure 5:**
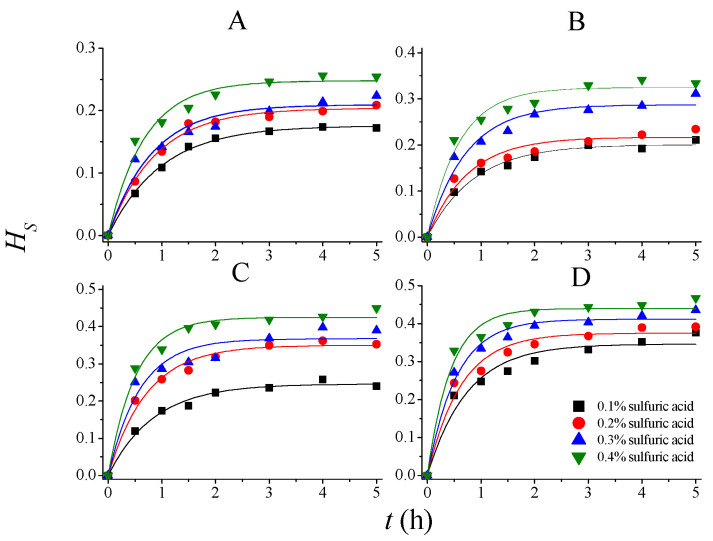
Experimental and model-predicted kinetic data for holocellulose solubilization at different temperatures. (**A**): 80 °C; (**B**): 90 °C; (**C**): 100 °C; and (**D**): 110 °C.

The rate constants and parameters for PDR can thus be determined according to experimental results. The experimental and model predicted data are shown in Figure 3, Figure 4 and Figure 5, and the corresponding regressed rate constants (*k*, including *k_S_*, *k_L_*_,_ and *k_H_*) and parameters for potential degree of reaction (*δ*, including *δ_SS_*, *δ_DD_*, and *δ_HS_*) are shown in Table 2. It is clear that the PDR model demonstrated much higher accuracy than Saeman’s model to describe the kinetics of *SS*, *DD*, and *HS* during 75% AA pretreatment. The determination coefficients of the models are in the range of 0.95–0.99, indicating that the model fitted the experimental results very well.

Both *δ* and *k* increased with *C_SA_* and temperature. This is because the fragmentation of lignin and degradation of polysaccharides can be facilitated by acid-catalysts. The fragmentation of lignin during organosolv pretreatment is mainly attributed to the cleavage of ether linkages. In acidic systems, easily hydrolysable α-ether linkages are most readily broken. However, cleavage of β-aryl ether bonds also takes place, and this may be more important than cleavage of α-ether linkages for lignin fragmentation, especially under strong acid system [25]. The effect of *T* on the reaction rate can be described by the Arrhenius equation. In order to correlate *k* or *δ* with *C_SA_* and *T*, an extended Arrhenius equation or modified logistic equation is used as follows, respectively, according to the work of Dong et al. [24].

(17) $$k=k_{0}\exp(-\frac{E_{a}}{RT})C_{SA}^{\alpha }$$

(18) $$\delta =1-\frac{1}{1+AC_{SA}^{m}R_{0}^{n}}$$

where; *k*_0_ and *E_a_* are pre-exponential factor and activation energy, respectively; *R*_0_ is the temperature-dependent severity factor ($R_{0}=\exp(\frac{T^{\prime }-100}{14.75})$, where *T*′ is reaction temperature in unit of °C), *A* is an adjustable parameter, and *m* and *n* are corresponding order parameters for *C_SA_* and *R*_0_. Equations (17) and (18) can be expressed as:

(19) $$\ln k=\ln k_{0}-\frac{E_{a}}{RT}+\alpha \ln C_{SA}^{}$$

(20) $$\ln\left(\frac{1}{1-\delta }-1\right)=\ln A+m\ln C_{SA}+n\ln R_{0}$$

**Figure 6:**
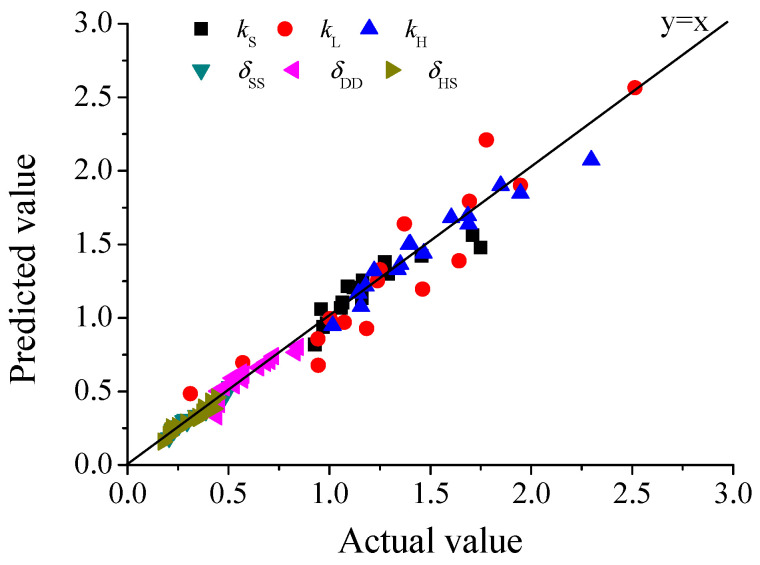
Comparison of actual values and model predicted values for *k* and *δ*.

Therefore, based on the data listed in Table 2, corresponding kinetic constants could be determined by multiple linear regression, and the results are shown in Table 3. Corresponding comparison of actual values (shown in Table 2) and regressed model-predicted values for *k* and *δ* are shown in Figure 6. The results demonstrated that Equations (17) or (18) could well correlate the relation of *k* or *δ* with *C_SA_* and *T*. Therefore, the *S_S_*, *D_d_* and *H_S_* can be calculated by the following integral kinetic models:

(21) $$S_{S}=(1-\frac{1}{1+3.2135C_{SA}^{0.4284}R_{0}^{0.5011}})\{1-\exp[-343.52\exp(-\frac{14573}{RT})C_{SA}^{0.2348}t]\}$$

(22) $$D_{d}=(1-\frac{1}{1+30.3386C_{SA}^{0.7421}R_{0}^{0.5281}})\{1-\exp[-9.3887\times 10^{5}\exp(-\frac{35533}{RT})C_{SA}^{0.5176}t]\}$$

(23) $$H_{S}=(1-\frac{1}{1+2.2262C_{SA}^{0.4003}R_{0}^{0.4619}})\{1-\exp[-393.52\exp(-\frac{13636}{RT})C_{SA}^{0.3021}t]\}$$

The results demonstrated that the activation energy for delignification under the experimental condition (75% AA) was 35.533 kJ/mol, which was much lower than that reported by other researchers [15,26,27]. However, it should be noted that the activation energy determined in the present work was the apparent activation energy using 75% AA for delignification. If the contribution of AA was excluded, the determined activation energy (intrinsic activation energy) should be higher. The experimental results also indicated that the *S_S_* and *H_S_* had smaller activation energies, indicating that they are less sensitive to temperature. Actually, holocellulose contains cellulose and hemicellulose, and hemicelluloses are much easier than cellulose to degrade during AA pretreatment. It has been found that, under the used severest pretreatment conditions (0.0408 mol/L SA and 90% AA), *S_S_* reached about 57%, with both *D_d_* and xylan solubilization being higher than 90%, while cellulose solubilization was less than 25%. Therefore, because cellulose was much inerter than lignin, the *k* and *δ* for *H_S_* were apparently smaller than those of *D_d_*.

### 2.3. Effects of AA Pretreatment on the Enzymatic Hydrolysis of Pretreated Solid

**Figure 7:**
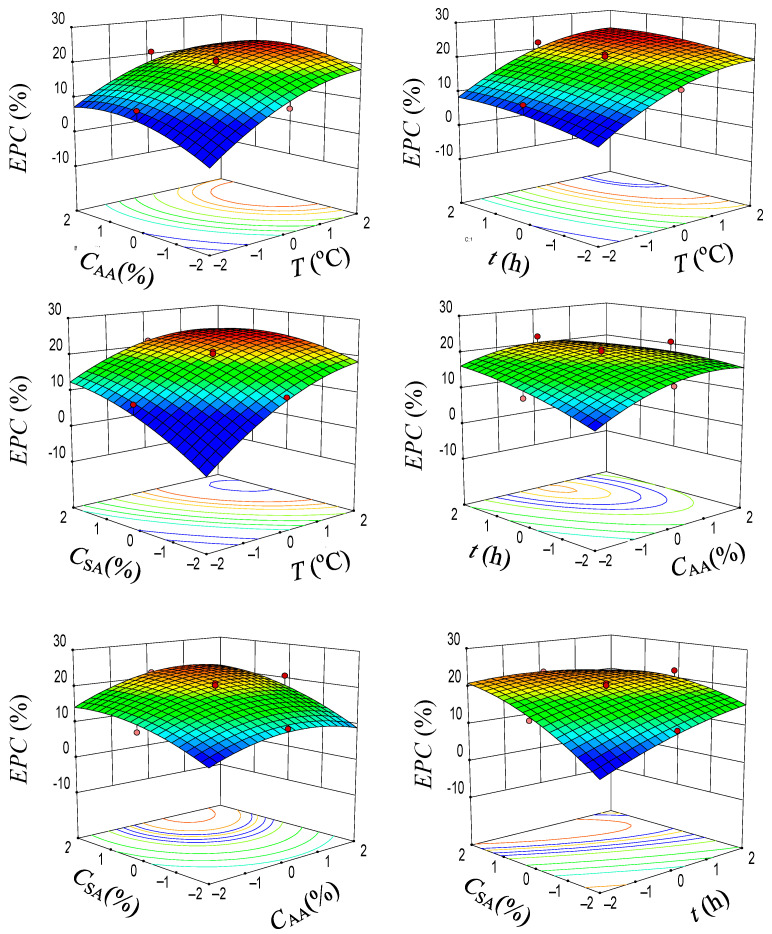
Three-dimensional surface plots of effects of temperature (*T*, °C), AA concentration (*C_AA_*, wt%), pretreatment time (*t*, h), and sulfuric acid concentration (*C_SA_*, wt%) on enzymatic polysaccharide conversion (*EPC*, %) of AA-pretreated substrates. The red color indicates a high level and the blue indicates a low level.

The CCD experimental results for effects of different parameters in AA pretreatment on enzymatic polysaccharide conversion (EPC) are shown in Table 1. Corresponding statistical analysis results are shown in Table 4. *T* and *C_SA_* showed very significant effects (*p* < 0.01) on *EPC*, while *C_AA_* and *t* showed significant effects (*p* < 0.05). The interactive effects of these variables showed no significant effects (*p* > 0.05), but the effects of quadric terms of *T* and *C_AA_* were significant (*p* > 0.05), indicating that *T* and *C_AA_* demonstrated significant non-linear effects on *EPC*. From the three-dimensional surface plots (Figure 7), it can be known that *EPC* increased with *T*, *t*, and *C_SA_* continuously. However, the effect of *C_AA_* showed a parabolic tendency with the maximum *EPC* obtained at around 75%. Further increase in *C_AA_* oppositely decreased *EPC*. This was mainly because the severer acetylation of cellulosic solid took place at higher *C_AA_*, leading to weakening of cellulose recognition by cellulase enzymes [18]. Therefore, based on the above optimization and kinetic results, AA concentration should be controlled at about 75% in order to obtain a compromised optimum *EPC*.

### 2.4. Effect of PAA Post-Treatment on Cellulose Hydrolysis

Table 1 showed that the obtained *D_d_* was generally in the range of 20–70%, mainly depending on the AA concentration and reaction temperature, which increased the cellulose enzymatic digestibility to some extents. However, the *EPC* was still very low (less than 30%). This was mainly because the *D_d_* was not high enough. AA-pretreatment was conducted under a relatively mild condition (<110 °C), and such a mild condition could not cause a significant modification of lignin structure and migration of lignin in the cell wall layers. Therefore, in order to obtain a greatly improved cellulose digestibility, a high *D_d_* should be obtained. Moreover, the acetyl group introduced in AA-pretreatment also inhibited enzymatic hydrolysis. Therefore, it is necessary to remove the acetyl group by post-treatment. Previous works have demonstrated that alkalis, such as NaOH, Ca(OH)_2_, or NH_4_OH, are good reagents for deacetylation [28]. However, water washing is needed prior to alkaline deacetylation. In the present work, we employed peracetic acid (PAA) for post-treatment at PAA loading (based on initial dry bagasse weight) of 2.5–10%, which not only removed acetyl group, but also selectively removed lignin by oxidative degradation. The chemical compositions of PAA post-treated substrates are shown in Table 5. As expected, both further delignification and deacetylation were achieved by PAA post-treatment. The residual lignin content could be reduced by about 50%, while the degradation of hemicelluloses (xylan) was less than 20%, indicating that PAA was very selective towards delignification. For example, after sugarcane bagasse was pretreated by 70% AA with 0.3% SA at 110 °C for 2 h, followed by 10% PAA post-treatment, the lignin content could be reduced to 5.88%. However, to achieve a similar degree of delignification by single-stage PAA treatment, PAA loading should be higher than 40% [29].

**Figure 8:**
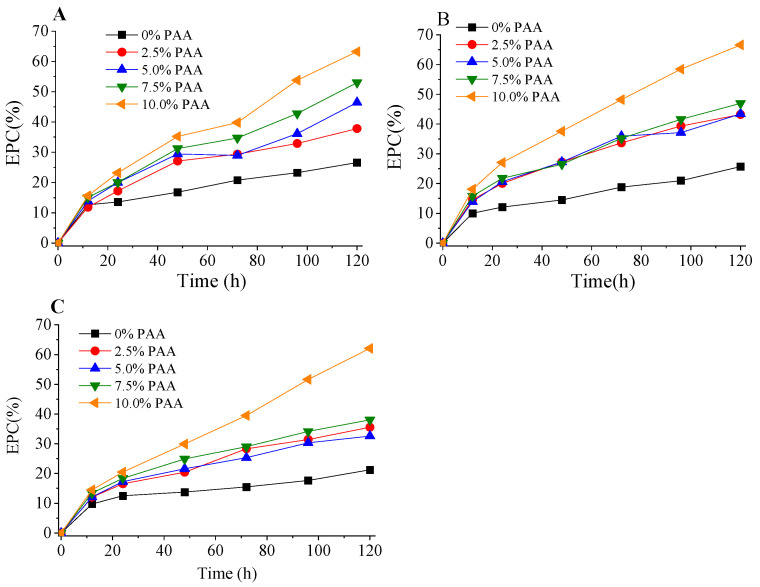
Enzymatic hydrolysis of PAA post-treated substrates. (**A**) 60% AA pretreatment; (**B**) 70% AA pretreatment; (**C**) 80% AA pretreatment.

The acetyl group of the AA-pretreated solid could be reduced from 2.4–3.4%, depending on AA pretreatment to less than 1% when 10% PAA loading (based on initial dry bagasse weight) was used. The enzymatic hydrolysis of PAA post-treated substrates (Figure 8) illustrated that PAA post-treatment could dramatically increase the *EPC* of the substrates. The highest *EPC* at 120 h was achieved with 70% AA pretreatment and 10% PAA post-treatment, reaching about 70%, compared with only 26% of the control (without PAA post-treatment). This was mainly because PAA post-treatment significantly removed the residual lignin, as well as the acetyl group, causing a high degree of delignification (>85%), with an associated liberation of cellulose fibers and an increase in the hydrophilicity of the substrates. Moreover, it has been known that cellulase enzymes could be non-productively adsorbed on the residual lignin by hydrophobic, hydrogen bonding, electrostatic interactions, and cation−π interactions [30]. Oxidative pretreatments, such as by PAA delignification, could well modify lignin structure to increase its hydrophilicity, thus reducing the non-productive adsorption of enzymes on lignin [20]. However, the mechanism on the improved digestibility has to be further investigated in terms of the cell wall microstructure changes and surface characteristics of the substrates. However, it should be noted that PAA post-treatment could be performed without water washing of the AA pretreated solid, and the decomposition of PAA is mainly water and oxygen, which is involve a green process without pollutants. Hence, compared with alkali-PAA pretreatment, the AA-PAA process would require less water and PAA consumption. It also should be noted that the cellulase used in the present work was not a specific enzyme formula for lignocellulose hydrolysis. Thus, the sub-enzyme components of the cellulase complex were not optimal for biomass degradation. Therefore, detailed comparison of the results of the present work with those reported by the literature is not possible. However, according to Zhao and Liu [19], when bagasse was pretreated, first, by 80–90% AA, followed by 1–4% NaOH (based on initial bagasse weight) deacetylation, the enzymatic glucan conversions with 20 FPU/solid of cellulase complex (Novozym Celluclast 1.5 L, specific cellulase for biomass degradation) and 40 CBU/g solid of supplemental β-glucosidase were in the range of 60–90%. The *EPC* obtained in the present work was in this range.

## 3. Materials and Methods

### 3.1. Materials

The sugarcane bagasse used in the present work was collected in Guangxi Zhuang Autonomous Region in South China. It was ground and screened, and the fraction retained by a 20-mesh sieve was used in all pretreatment experiments. The main chemical compositions of the bagasse were determined to be 5.02% moisture, 71.26% holocellulose, 43.68% cellulose, 27.58% hemicellulose,19.25% acid-insoluble lignin, and 1.90% acid-soluble lignin. The cellulase enzymes used for enzymatic hydrolysis of the pretreated cellulosic solid was Cellulase R-10 produced by Yakuh Honsha Co. Ltd. (Tokyo, Japan), with a filter paper activity of ~6000 FPU/g enzyme powder. The chemical agents used in the pretreatments, including acetic acid (AA), H_2_O_2_, and sulfuric acid, were purchased from Beijing Beihua Fine Chemicals Co., Ltd. (Beijing, China). Peracetic acid (PAA) was prepared by reaction of AA and 30 wt% H_2_O_2_, with a volume ratio of 2.5:1 at room temperature for 72 h. 3% (*w*/*w*) sulfuric acid was added as a catalyst, according to our previous kinetic modeling and optimization works [31,32,33]. The obtained PAA solution had a PAA concentration of about 2.2 mol/L. It should be noted that PAA is not stable and easy to decompose. Therefore, in order to ensure its purity and concentration, PAA solution was prepared before experiments and stored in fridge. Moreover, before preparation, the vessels used were carefully washed to avoid contamination by metal ions that can catalyze the decomposition of peracetic acid. The standard glucose, xylose, and arabinose were purchased from Sigma-Aldrich (Shanghai agent, China).

### 3.2. AA Pretreatment and PAA Post-Treatment

AA pretreatment was carried out in a 1000 mL three-neck glass flask heated by water bath or electric jacket under atmospheric pressure. An amount of 30 g of screened bagasse was packed into the flask followed by addition of 300 mL 60–90 wt.% AA solution with 0–0.5 wt.% sulfuric acid (in liquid phase). Electrical stirring with a Teflon paddle was used at 300 rpm to keep the system as homogeneous as possible. After AA pretreatment, the mixture was filtered using a Buchner funnel. The obtained solid was first washed with 300 mL 60–90 wt% AA solution and then filtered to remove as much liquid as possible. Typically, after filtration, the liquid content of the pretreated solid was 75–80%. When no PAA post-treatment was performed, the filtered solid was washed by running water until neutrality and filtered and oven-dried for further analysis of chemical compositions.

PAA post-treatment was carried out in a 1000 mL glass flask immersed in a water bath at 75 °C. The AA-washed and filtered pretreated solid was packed into the flask, and a certain volume of prepared PAA solution was directly added with PAA loading of 0–10% (based on initial dry bagasse weight before AA pretreatment). A Teflon^®^ paddle was used for intermittently stirring to keep the system as uniform as possible. After PAA post-treatment, the solid was washed using running water until neutrality, and then it was filtered and oven-dried for further analysis of chemical compositions.

### 3.3. Enzymatic Hydrolysis of Pretrereated and Post-Treated Substrates

The AA-pretreated and PAA-post-treated substrates were digested by cellulase loading of 20 FPU/g solid at temperature 50 ± 0.5 °C, pH 4.8 (0.1 mol/L sodium acetate buffer), and 130 rpm in an air-bath shaker. Enzymatic digestibility, denoted as enzymatic polysaccharide conversion (*EPC*, %), was defined as the percentage of holocellulose converted to reducing sugar (glucose plus xylose) after incubation with cellulase enzyme.

### 3.4. Experimental Design

To optimize the AA pretreatment process, a response surface methodology (central composite design, CCD) was employed to study the effects of temperature (*T*, °C), AA concentration (*C_AA_*, wt%), pretreatment time (*t*, h), and sulfuric acid concentration (*C_SA_*, wt% or mol/L) on several response variables, including solid recovery yield (*SY*, %), degree of delignification (*DD*, %), solubilization of holocellulose (*HS*), acetyl group content (*AGC*, %), and enzymatic polysaccharide conversion (*EPC*, %). The levels of the variables are summarized in Table 6. A CCD with eight star points, as well as six replicates at the center points, leading to 30 runs, was employed for the optimization. The variables were coded according to the following equation:

(24) $$X_{i}=\frac{x_{i}-x_{0}}{\Delta x},\;i=1,\;2\ldots k$$

where; *X_i_* is the dimensionless value of an variable; *x_i_* is the real value of an variable; *x*_0_ is the level value of *X_i_* at the center point; and Δ*x* is the step change. A quadratic polynomial equation (Equation (25)), including all interaction terms, was used to calculate the predicted response variable.

(25) $$Y_{i}=\beta_{0}+\sum_{i=1}^{4}\beta_{i}X_{i}+\sum_{i=1}^{4}\beta_{ii}X_{i}^{2}+\sum_{i,j=1}^{4}\beta_{ij}X_{i}X_{j}$$

where; *Y_i_* is the predicted response variable; *X_i_* and *X_j_* are the input variables; *β*_0_ is the intercept term; *β_i_*, *β_ii_*, and *β_ij_* are the regressed parameters for linear effects, squared effects (non-linear effects), and interactive effects, respectively. Design-Expert 9.0.6 software 8.0.7.1 (Stat-Ease, Inc., Minneapolis, MN, USA) was used to make the experimental design, regress the parameters, and make statistical analysis. The experimental design is shown in Table 6.

### 3.5. Analytic Methods

The chemical compositions of bagasse and pretreated substrates were analyzed in accordance with corresponding Chinese Standards, namely, moisture content, GB/T 2677.2–1993, ash, GB/T 2677.3–1993, hot water extractives, GB/T 2677.4–1993, 1% NaOH extractives, GB/T 2677.5–1993, benzene-ethanol extractives, GB/T 2677.6–1994, holocellulose, GB/T 2677.10–1995, Klason lignin, GB/T 2677.8–1994, and acid-soluble lignin, GB/T 747–2003. The cellulose content was measured by the nitric acid–ethanol method [34]. The determination of PAA concentration was in accordance with Chinese standard GB 19104–2008. The monosaccharides and ethanol were determined by Shimadzu (Tokyo, Japan) HPLC (LC-10AT) equipped with a SCL-10A system controller, a CTO-AS column oven, a RID-10A refractive index detector, and an Aminex HPX-87H column. The mobile phase was 0.005 M H_2_SO_4_ at a flow rate of 0.8 mL/min. The cellulase activity (filter paper activity) was determined according to Ghose [35], but, this was performed by using HPLC to determine the formed glucose concentration instead of using 3,5-dinitrosalicylic acid to measure the reducing sugar concentration.

## 4. Conclusions

A response surface methodology (central composite design, CCD) was employed to study the effects of several factors on pretreatment of sugarcane bagasse (SCB) by aqueous acetic acid (AA) with addition of sulfuric acid (SA) as a catalyst under mild condition (<110 °C). Several quadratic polynomial models were obtained, based on the CCD experimental results. As found in the experiments, temperature, AA concentration, time, and SA concentration showed significant effects on solid yield (*SY*), degree of delignification (*DD*), holocellulose solubilization (*HS*), acetyl group content (*AGC*), and enzymatic polysaccharide conversion (*EPC*). *SY*, *DE*, and *HS* were increased in relation to the levels of the factors. However, higher *AGC* was observed at high AA concentration because of cellulose acetylation. Kinetic modeling was further investigated for AA pretreatment using both Saeman’s model and the potential degree of reaction (*PDR*) model. However, Saeman’s model showed a great deviation from the experimental results, while the PDR model fit the experimental data very well, with determination coefficients of 0.95–0.99. Nevertheless, the enzymatic digestibility of the AA-pretreated substrates was still lower than 30%, mainly due to the relatively low degree of delignification and acetylation of cellulose. Post-treatment of the pretreated cellulosic solid by PAA with loading of 10% (based on initial dry sugarcane bagasse weight) well improved the cellulose digestibly by further selectively removing 50–60% of the residual linin and acetyl group. The enzymatic polysaccharide conversion increased from <30% for AA-pretreatment to about 70% for PAA post-treatment. Compared with alkaline deacetylation, PAA post-treatment avoided the water washing step after AA pretreatment.

## Figures and Tables

**Table 1:** CCD experimental design and results of AA pretreatment of bagasse.

Run	Variables				*SY* (%)		*DD* (%)		*HS* (%)		*AGC* (%)		*EPC* (%)
	*X*_1_	*X*_2_	*X*_3_	*X*_4_	Exp.	Pred.	Exp.	Pred.	Exp.	Pred.	Exp.	Pred.	Exp.
1	−1	−1	−1	−1	93.30	91.40	24.77	28.11	3.01	2.01	4.60	4.53	7.08
2	1	−1	−1	−1	82.13	84.46	34.88	31.81	14.10	11.71	4.44	4.52	14.43
3	−1	1	−1	−1	92.10	89.06	25.70	29.51	3.44	7.61	4.80	4.78	6.76
4	1	1	−1	−1	78.50	77.12	40.88	43.61	19.82	20.71	4.74	4.86	18.34
5	−1	−1	1	−1	91.35	88.28	23.23	24.73	4.57	6.75	4.40	4.52	8.95
6	1	−1	1	−1	78.00	78.26	21.02	27.87	20.31	19.81	4.54	4.32	21.44
7	−1	1	1	−1	88.20	85.98	28.06	29.61	5.75	10.07	5.05	4.93	9.05
8	1	1	1	−1	75.80	70.96	40.65	43.15	24.29	26.53	4.76	4.83	21.16
9	−1	−1	−1	1	81.00	84.90	31.04	28.73	12.89	11.33	4.70	4.75	10.73
10	1	−1	−1	1	71.85	72.84	47.17	49.87	27.34	24.07	4.25	4.13	23.43
11	−1	1	−1	1	78.60	77.12	38.17	35.61	18.28	19.85	5.25	5.24	17.07
12	1	1	−1	1	57.88	60.06	68.43	67.15	37.48	35.99	4.74	4.72	23.36
13	−1	−1	1	1	81.00	81.14	30.99	32.55	17.45	17.63	4.83	4.49	14.72
14	1	−1	1	1	63.90	66.00	56.75	53.13	37.18	33.73	3.57	3.69	22.47
15	−1	1	1	1	76.65	73.40	39.62	42.91	20.80	23.87	5.10	5.14	16.8
16	1	1	1	1	52.60	53.26	72.96	73.89	41.31	43.37	4.61	4.44	21.68
17	−2	0	0	0	81.60	85.98	33.45	30.60	14.03	8.41	4.79	4.90	9.95
18	2	0	0	0	61.10	58.90	66.95	65.28	33.76	37.61	4.15	4.18	21.31
19	0	−2	0	0	88.35	84.90	28.99	27.74	8.65	14.43	4.36	4.53	10.87
20	0	2	0	0	64.20	69.82	53.14	49.90	37.23	29.67	5.56	5.53	20.67
21	0	0	−2	0	87.48	85.60	36.14	36.66	8.74	11.15	4.97	4.91	14.33
22	0	0	2	0	71.64	75.68	45.06	40.02	27.41	23.27	4.41	4.61	22.00
23	0	0	0	−2	84.40	90.22	27.14	19.78	7.51	3.41	4.28	4.24	12.10
24	0	0	0	2	69.70	66.02	48.30	51.14	27.21	29.57	3.90	4.06	21.44
25	0	0	0	0	77.40	79.28	37.09	36.74	23.36	22.61	4.56	4.91	17.89
26	0	0	0	0	80.88	79.28	36.55	36.74	21.09	22.61	5.28	4.91	20.43
27	0	0	0	0	79.85	79.28	36.40	36.74	22.56	22.61	4.79	4.91	21.05
28	0	0	0	0	78.92	79.28	37.01	36.74	23.12	22.61	4.86	4.91	18.49
29	0	0	0	0	80.10	79.28	36.42	36.74	21.55	22.61	4.92	4.91	17.01
30	0	0	0	0	78.54	79.28	36.98	36.74	22.98	22.61	5.02	4.91	20.51

**Table 2:** Determined parameters for *SS*, *DD*, and *HS* by the PDR model, according to experimental data, with 75% AA.

*T* (°C)	*C_SA_* (mol/L)							
	0.0102		0.0204		0.0306		0.0408	
	*δ*	*k* (h^−1^)	*δ*	*k* (h^−1^)	*δ*	*k* (h^−1^)	*δ*	*k* (h^−1^)
For *SS*								
80	0.2063	0.9280	0.2297	0.9860	0.2543	0.9598	0.2951	1.1617
90	0.2239	0.9684	0.2790	1.0650	0.3413	1.0901	0.3884	1.2900
100	0.2876	1.0570	0.3924	1.1641	0.4367	1.2756	0.4726	1.7496
110	0.4207	1.1189	0.4625	1.4572	0.4819	1.7093	0.5092	2.5670
For *DD*								
80	0.4437	0.3116	0.4548	0.5714	0.4794	0.9436	0.5716	1.0047
90	0.4567	0.9447	0.5297	1.0737	0.5760	1.4615	0.6493	1.6422
100	0.4485	1.1850	0.5663	1.2515	0.6844	1.3721	0.7232	1.9464
110	0.5190	1.2391	0.7063	1.6942	0.8315	1.7779	0.8473	2.5142
For *HS*								
80	0.1759	1.0153	0.2039	1.1438	0.2093	1.2217	0.2477	1.4665
90	0.2007	1.1562	0.2170	1.3381	0.2874	1.4000	0.3246	1.6903
100	0.2460	1.1801	0.3499	1.3930	0.3674	1.6862	0.4250	1.9468
110	0.3462	1.3510	0.3749	1.6038	0.4118	1.8492	0.4400	2.2970

**Table 3:** Determination of *k* and *δ* by multivariate linear regression.

***k***	***k*_0_****	***E_a_* (kJ/mol)**	***α***	***R*^2^****	***F***	**P**
*k_S_*	343.52	14.573	0.2348	0.8272	28.7148	0.0000
*k_L_*	9.3887 × 10^5^	35.533	0.5176	0.8448	35.3855	0.0000
*k_H_*	393.52	13.636	0.3021	0.9386	99.3466	0.0000
***δ***	***A***	***m***	***n***	***R*^2^****	***F***	***P***
*δ_SS_*	3.2135	0.4284	0.5011	0.9627	167.9312	0.0000
*δ_DD_*	30.3386	0.7421	0.5281	0.8595	39.7707	0.0000
*δ_HS_*	2.2262	0.4003	0.4619	0.9434	108.4232	0.0000

**Table 4:** ANOVA for the response surface quadratic model for enzymatic polysaccharide conversion.

Source	Sum of Squares	df	Mean Squares	*F* Value	*p*-Value *p* > *F*
Model	713.04	14	50.93	9.57	<0.0001
*X*_1_	399.11	1	399.11	74.99	<0.0001
*X*_2_	38.94	1	38.94	7.32	0.0163
*X*_3_	38.53	1	38.53	7.24	0.0168
*X*_4_	158.77	1	158.77	29.83	<0.0001
*X*_1_ *X*_2_	1.84	1	1.84	0.35	0.5650
*X*_1_ *X*_3_	0.030	1	0.030	0.0059	0.9414
*X*_1_ *X*_4_	8.87	1	8.87	1.67	0.2164
*X*_2_ *X*_3_	4.79	1	4.79	0.90	0.3580
*X*_2_ *X*_4_	1.08	1	1.08	0.20	0.6593
*X*_3_ *X*_4_	10.42	1	10.42	1.96	0.1821
*X*_1_^2^	26.45	1	26.45	4.97	0.0415
*X*_2_^2^	24.60	1	24.60	4.62	0.0483
*X*_3_^2^	3.33	1	3.33	0.62	0.4415
*X*_4_^2^	13.32	1	13.32	2.50	0.1344
Residual	79.83	15	5.32		
Lack of Fit	66.17	10	6.62	2.42	0.1705
Pure Error	13.66	5	2.73		
Cor Total	792.87	29			

**Table 5:** Chemical compositions of PAA post-treated substrates.

AA Pretreatment	PAA Loading (%) ^a^	*SY* (%)	Holocellulose (%)	Cellulose (%)	Xylan (%)	Total Lignin (%)	*AGC* (%)
60% AA, 0.3% SA, 110 °C, 2 h	0	59.8	86.0	62.6	16.1	14.7	2.46
	2.5	57.6	88.9	67.5	15.9	12.1	1.87
	5.0	55.4	90.1	68.6	16.8	10.5	1.23
	7.5	53.1	90.9	70.2	17.0	8.42	0.78
	10	50.1	92.3	72.5	17.4	6.23	0.58
70% AA, 0.3% SA, 110 °C, 2 h	0	54.0	88.2	65.6	15.8	13.8	3.15
	2.5	52.1	89.5	68.9	15.7	10.2	2.59
	5.0	50.3	90.6	70.4	16.4	8.31	1.58
	7.5	49.7	92.2	74.3	15.9	6.18	1.22
	10	48.9	93.1	75.6	16.6	5.88	0.69
80% AA, 0.3% SA, 110 °C, 2 h	0	52.1	90.1	68.2	15.6	10.2	3.45
	2.5	50.5	92.4	70.6	16.6	8.27	3.00
	5.0	48.7	93.2	73.4	16.0	7.01	2.45
	7.5	47.2	94.0	76.5	15.4	6.23	1.66
	10	46.7	94.4	77.7	15.9	5.12	0.96

^a^ based on initial dry bagasse weight.

**Table 6:** Levels of variables used in the CCD experimental design.

Variables, Abbreviation and Units	Code	Levels				
		−2	−1	0	1	2
Temperature (*T*, °C)	*X*_1_	70	80	90	100	110
AA concentration (*C_AA_*, wt%)	*X*_2_	55	65	75	85	95
Pretreatment time (*t*, h)	*X*_3_	1.0	1.5	2.0	2.5	3.0
Sulfuric acid concentration (*C_SA_*, wt%)	*X*_4_	0.0	0.1	0.2	0.3	0.4

## Data Availability

The data presented in this study are available on request from the corresponding author.

## Supplementary Materials

The following supporting information can be downloaded at: https://www.mdpi.com/article/10.3390/molecules28124689/s1, Figures S1–S4: 3D surface plots of effects of temperature (*T*, °C), AA concentration (*C_AA_*, wt%), pretreatment time (*t*, h) and sulfuric acid concentration (*C_SA_*, wt%) on solid recovery yield (*SY*, %), degree of delignification (*DD*, %), solubilization of holocellulose (*HS*, %), and acetyl group content (*AGC*, %) of pretreated substrates, respectively. Figures S5–S7: Plots of ln(1 − *S_S_*), ln(1 − *D_d_*), and ln(1 − *H_S_*) with *t* at different temperatures, respectively. Tables S1–S4: ANOVA for Response Surface Quadratic model for *SY*, *DD*, *HS* and *AGC*, respectively.
