# Supplementary material for: Improving the Cellulose Enzymatic Digestibility of Sugarcane Bagasse by Atmospheric Acetic Acid Pretreatment and Peracetic Acid Post-Treatment
Source: Molecules. 2023 Jun 10;28(12):4689. doi: 10.3390/molecules28124689 (PMC10304256; doi:10.3390/molecules28124689)
Supplement: Supplementary file 1 [file molecules-28-04689-s001.zip › molecules-2427227-supplementary.pdf]

Supporting information for:
Improving the cellulose enzymatic digestibility of sugarcane bagasse by atmospheric acetic acid pretreatment and peracetic acid post-treatment

Yuchen Bai [1, 2], Mingke Tian [1, 2], Zhiwei Dai [3, 4] and Xuebing Zhao [3, 4,*]

1 China Food Flavor and Nutrition Health Innovation Center, Beijing Technology and Business University, Beijing 100048, China; baiyuchen@btbu.edu.cn (Y.B.)
2 Beijing Key Laboratory of Flavor Chemistry, Beijing Technology and Business University, Beijing 100048, China
3 Key Laboratory of Industrial Biocatalysis, Ministry of Education, Tsinghua University, BBeijing 100084, China
4 IInstitute of Applied Chemistry, Department of Chemical Engineering, Tsinghua University, Beijing 100084, China

* Correspondence: zhaoxb@mail.tsinghua.edu.cn

Figures

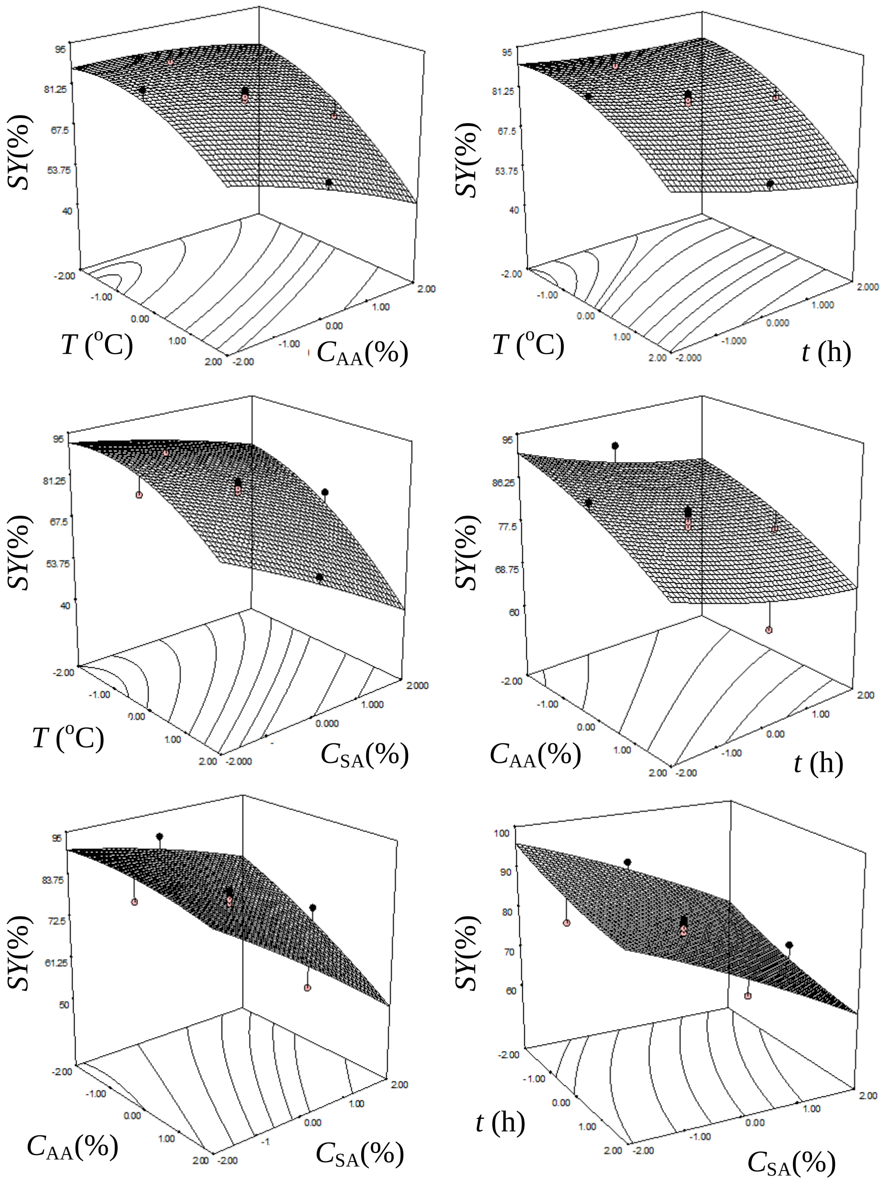

Figure S1. 3D surface plots of effects of temperature (T, °C), AA concentration (C_{AA}, wt%), pretreatment time (t, h) and sulfuric acid concentration (C_{SA}, wt%) on solid recovery yield (SY, %).

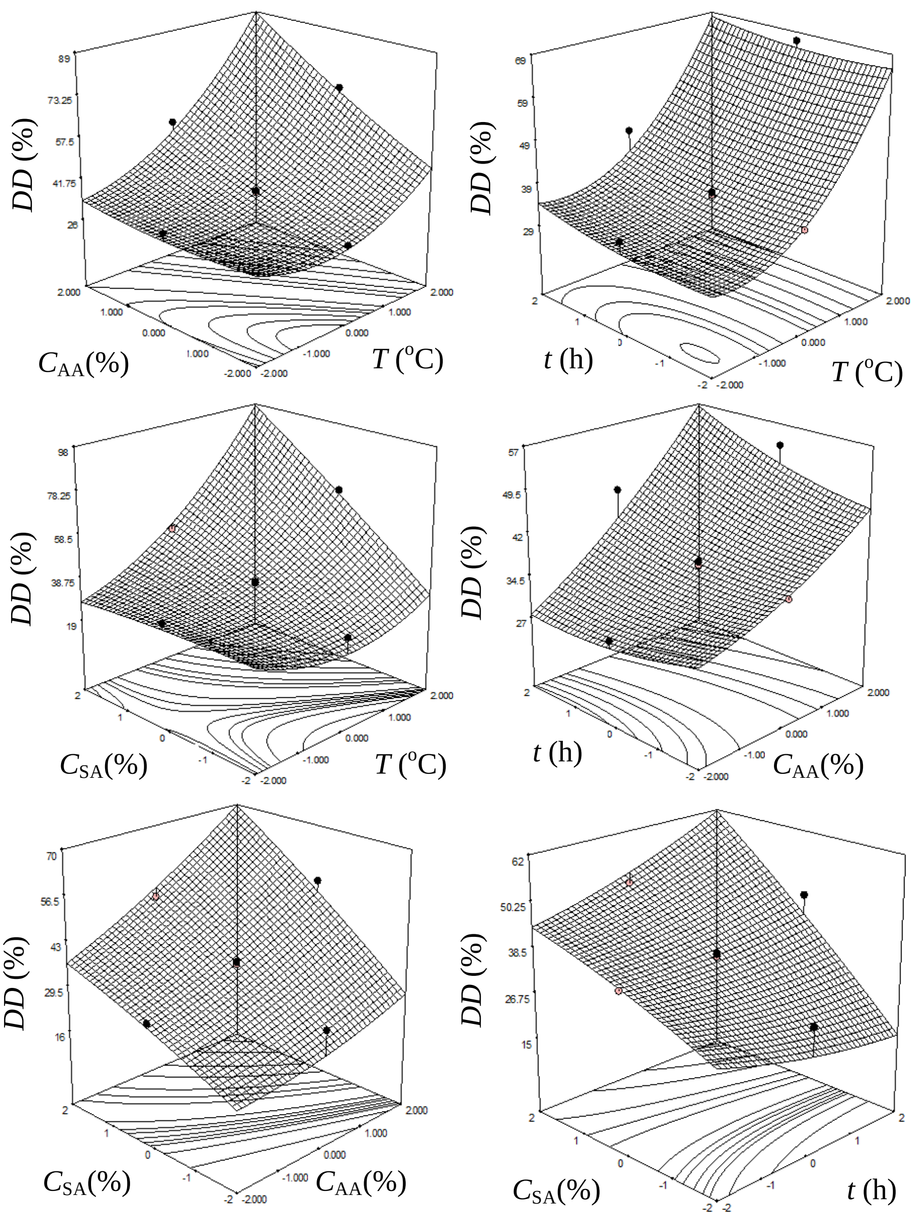

Figure S2. 3D surface plots of effects of temperature (T, ^{o}C), AA concentration (C_{AA}, wt%), pretreatment time (t, h) and sulfuric acid concentration (C_{SA}, wt%) on degree of delignification (DD, %).

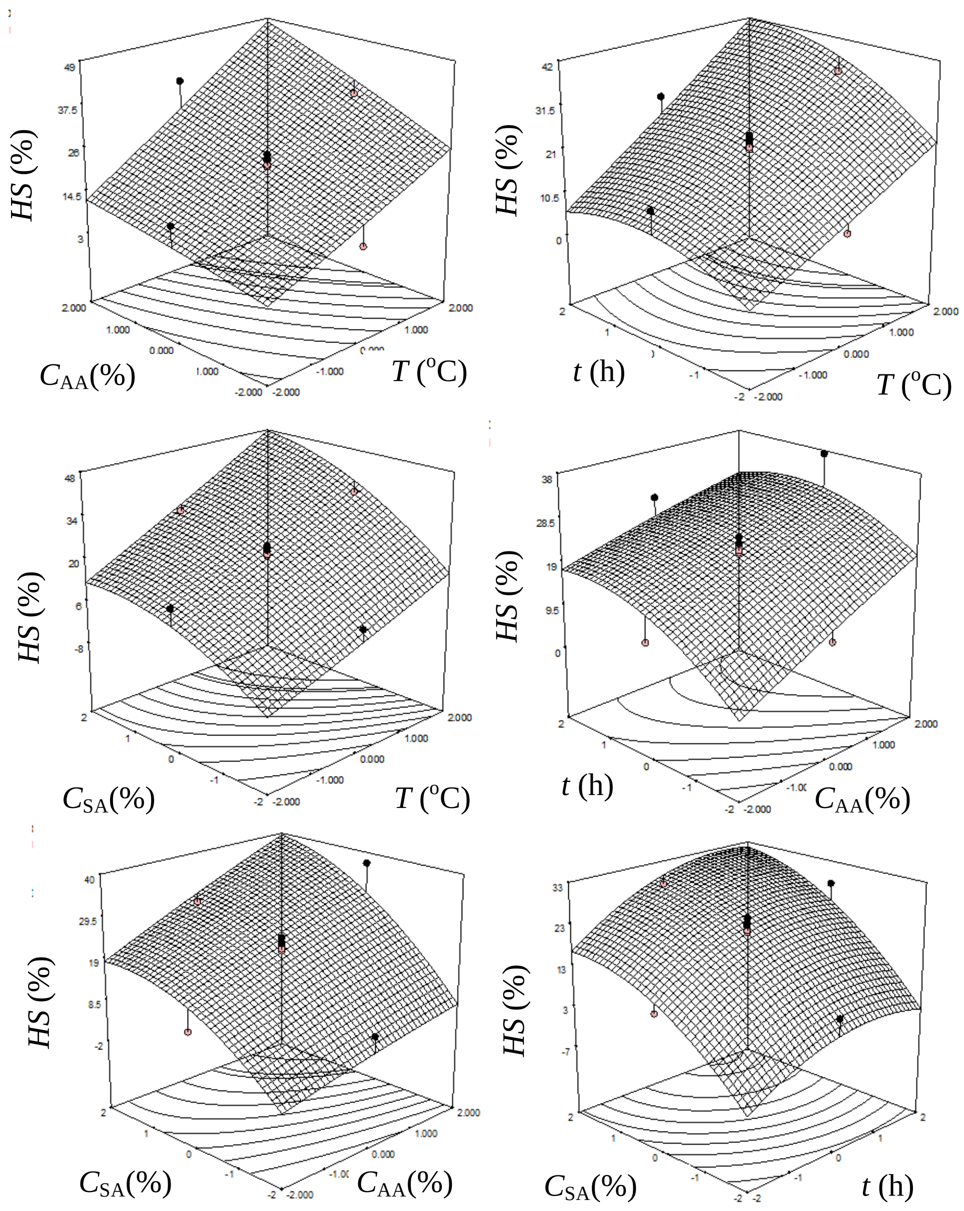

Figure S3. 3D surface plots of effects of temperature (*T*, °C), AA concentration (C_{AA}, wt%), pretreatment time (*t*, h) and sulfuric acid concentration (C_{SA}, wt %) on solubilization of holocellulose (*HS*, %).

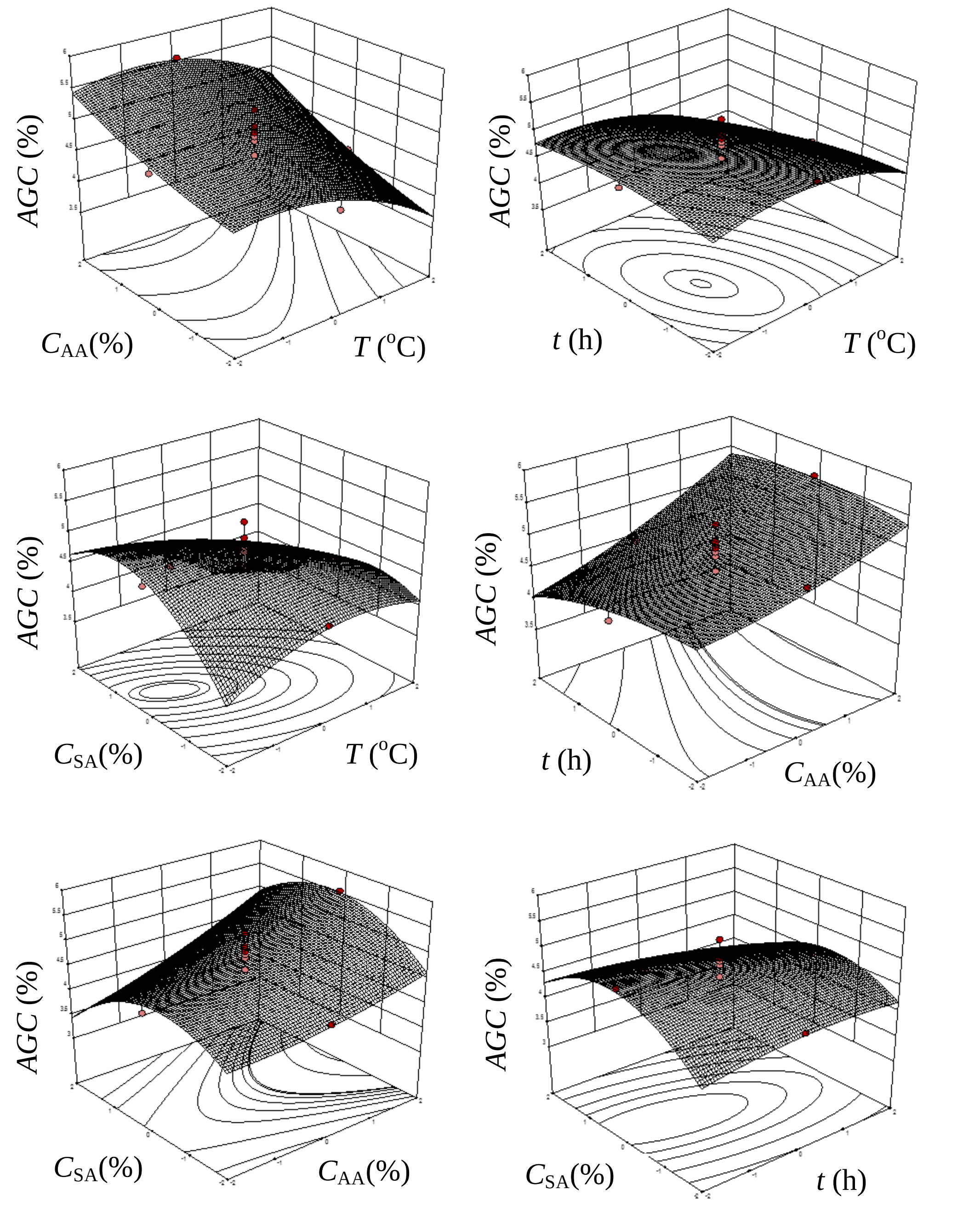

Figure S4. 3D surface plots of effects of temperature (*T*, °C), AA concentration (C_{AA}, wt%), pretreatment time (*t*, h) and sulfuric acid concentration (C_{SA}, wt%) on acetyl group content (*AGC*, %) of pretreated substrates.

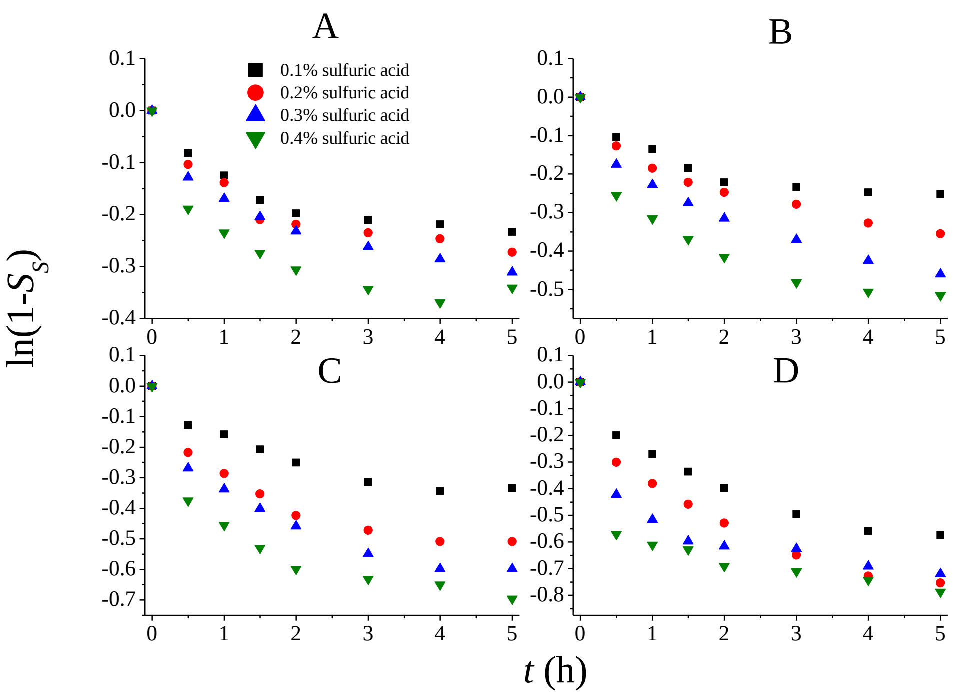

Figure S5. Plots of ln(1-S_S) with t at different temperatures. A: 80 °C； B: 90 °C； C: 100 °C； D: 110 °C.

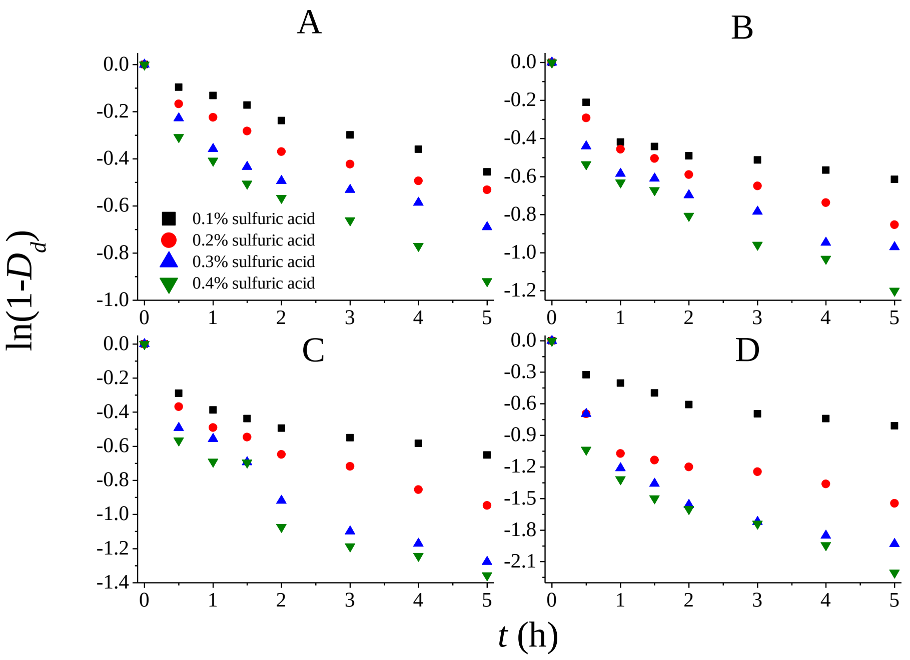

Figure S6. Plots of ln(1-D_d) with t at different temperatures. A: 80 °C； B: 90 °C； C: 100 °C； D: 110 °C.

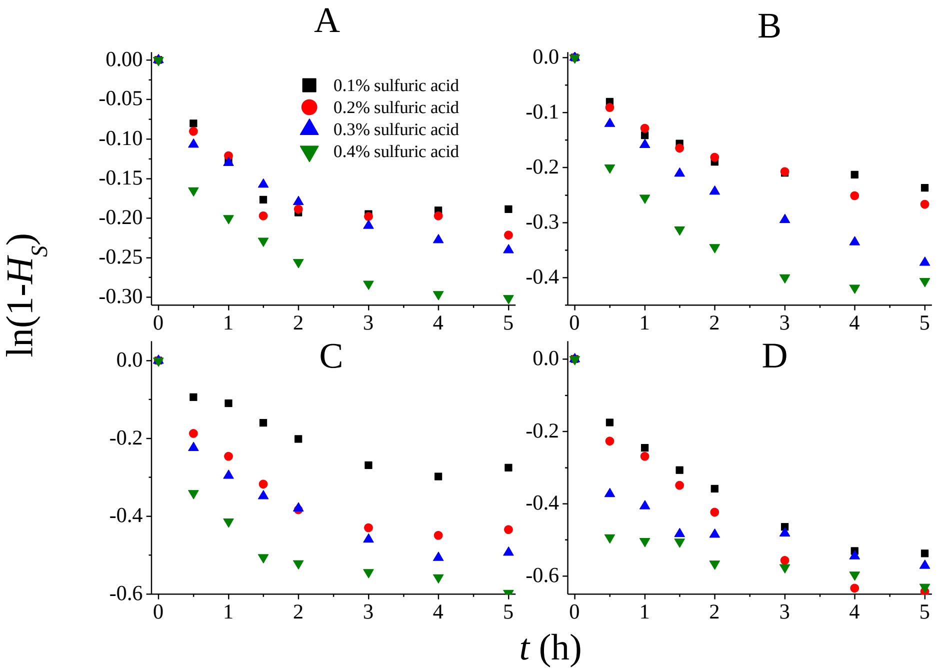

Figure S7. Plots of ln(1-*Hs*) with *t* at different temperatures. A: 80 °C; B: 90 °C; C: 100 °C; D: 110 °C.

Tables

Table S1. ANOVA for Response Surface Quadratic model for *SY*.

Source	Sum of Squares	df	Mean squares	*F* value	*P*-Value $P > F$
Model	2653.27	14	189.52	11.89	< 0.0001
X_1	1100.80	1	1100.80	69.05	< 0.0001
X_2	341.26	1	341.26	21.41	0.0003
X_3	147.71	1	147.71	9.26	0.0082
X_4	879.67	1	879.67	55.18	< 0.0001
X_1X_2	25.00	1	25.00	1.57	0.2297
X_1X_3	9.39	1	9.39	0.59	0.4546
X_1X_4	26.27	1	26.27	1.65	0.2188
X_2X_3	2.500E-003	1	2.500E-003	1.568E-004	0.9902
X_2X_4	29.81	1	29.81	1.87	0.1916
X_3X_4	0.39	1	0.39	0.025	0.8777
X_1^2	80.48	1	80.48	5.05	0.0401
X_2^2	6.36	1	6.36	0.40	0.5370
X_3^2	3.16	1	3.16	0.20	0.6624
X_4^2	2.27	1	2.27	0.14	0.7110
Residual	239.14	15	15.94		
Lack of Fit	231.37	10	23.14	14.89	0.0041
Pure Error	7.77	5	1.55		
Cor Total	2892.41	29			

Table S2. ANOVA for Response Surface Quadratic model for *DD*.

Source	Sum of Squares	df	Mean squares	F value	P-Value $P > F$
Model	4773.11	14	340.94	19.68	< 0.0001
X_1	1805.44	1	1805.44	104.24	< 0.0001
X_2	736.16	1	736.16	42.50	< 0.0001
X_3	16.80	1	16.80	0.97	0.3403
X_4	1476.74	1	1476.74	85.26	< 0.0001
X_1X_2	108.06	1	108.06	6.24	0.0246
X_1X_3	0.30	1	0.30	0.017	0.8966
X_1X_4	304.68	1	304.68	17.59	0.0008
X_2X_3	12.22	1	12.22	0.71	0.4142
X_2X_4	29.81	1	29.81	1.72	0.2093
X_3X_4	51.77	1	51.77	2.99	0.1044
X_1^2	215.33	1	215.33	12.43	0.0031
X_2^2	7.36	1	7.36	0.43	0.5243
X_3^2	4.43	1	4.43	0.26	0.6204
X_4^2	2.78	1	2.78	0.16	0.6946
Residual	259.80	15	17.32		
Lack of Fit	259.30	10	25.93	255.67	< 0.0001
Pure Error	0.51	5	0.10		
Cor Total	5032.91	29			

Table S3. ANOVA for Response Surface Quadratic model for *HS*.

Source	Sum of Squares	df	Mean squares	F value	P-Value $P > F$
Model	3021.88	14	215.85	11.42	< 0.0001
X_1	1277.50	1	1277.50	67.59	< 0.0001
X_2	348.69	1	348.69	18.45	0.0006
X_3	219.86	1	219.86	11.63	0.0039
X_4	1024.95	1	1024.95	54.23	< 0.0001
X_1X_2	11.59	1	11.59	0.61	0.4457
X_1X_3	11.22	1	11.22	0.59	0.4529
X_1X_4	9.21	1	9.21	0.49	0.4958
X_2X_3	5.11	1	5.11	0.27	0.6108
X_2X_4	8.56	1	8.56	0.45	0.5113
X_3X_4	2.40	1	2.40	0.13	0.7264
X_1^2	0.57	1	0.57	0.030	0.8650
X_2^2	0.25	1	0.25	0.013	0.9102
X_3^2	47.18	1	47.18	2.50	0.1350
X_4^2	60.91	1	60.91	3.22	0.0928
Residual	283.51	15	18.90		
Lack of Fit	279.28	10	27.93	33.02	0.0006
Pure Error	4.23	5	0.85		
Cor Total	3305.40	29			

Table S4. ANOVA for Response Surface Quadratic model for *AGC*.

Source	Sum of Squares	df	Mean squares	*F* value	*P*-Value $P > F$
Model	4.27	14	0.31	6.66	0.0004
X_1	0.79	1	0.79	17.28	0.0008
X_2	1.56	1	1.56	34.05	< 0.0001
X_3	0.13	1	0.13	2.88	0.1103
X_4	0.045	1	0.045	0.98	0.3371
X_1X_2	9.025E-003	1	9.025E-003	0.20	0.6635
X_1X_3	0.032	1	0.032	0.71	0.4136
X_1X_4	0.34	1	0.34	7.47	0.0154
X_2X_3	0.026	1	0.026	0.56	0.4664
X_2X_4	0.060	1	0.060	1.31	0.2704
X_3X_4	0.062	1	0.062	1.36	0.2611
X_1^2	0.24	1	0.24	5.14	0.0385
X_2^2	0.024	1	0.024	0.53	0.4773
X_3^2	0.039	1	0.039	0.85	0.3709
X_4^2	0.97	1	0.97	21.09	0.0004
Residual	0.69	15	0.046		
Lack of Fit	0.40	10	0.040	0.69	0.7105
Pure Error	0.29	5	0.058		
Cor Total	4.96	29			
